# Bazi Bushen attenuates osteoporosis in SAMP6 mice by regulating PI3K‐AKT and apoptosis pathways

**DOI:** 10.1111/jcmm.70161

**Published:** 2024-10-29

**Authors:** Zhe Xu, Zeyu Zhang, Huifang Zhou, Shan Lin, Boyang Gong, Zhaodong Li, Shuwu Zhao, Yunlong Hou, Yanfei Peng, Yuhong Bian

**Affiliations:** ^1^ School of Integrative Medicine Tianjin University of Traditional Chinese Medicine Tianjin P.R. China; ^2^ Graduate School Tianjin University of Traditional Chinese Medicine Tianjin P.R. China; ^3^ National Key laboratory of Luobing Research and Innovative Chinese Medicine Shijiazhuang P.R. China

**Keywords:** apoptosis, Bazi Bushen, bone homeostasis, bone mesenchymal stem cells, osteoporosis, PI3K‐AKT

## Abstract

Osteoporosis (OP), a systemic skeletal disease, is characterized by low bone mass, bone tissue degradation and bone microarchitecture disturbance. Bazi Bushen, a Chinese patented medicine, has been demonstrated to be effective in attenuating OP, but the pharmacological mechanism remains predominantly unclear. In this study, the senescence‐accelerated mouse prone 6 (SAMP6) model was used to explore bone homeostasis and treated intragastrically for 9 weeks with Bazi Bushen. In vivo experiments showed that Bazi Bushen treatment not only upregulated the levels of bone mineral density and bone mineral content but also increased the content of RUNX2 and OSX. Furthermore, the primary culture of bone mesenchymal stem cells (BMSCs) in SAMP6 mice was used to verify the effects of Bazi Bushen on the balance of differentiation between osteoblasts and adipocytes, as well as ROS and aging levels. Finally, the pharmacological mechanism of Bazi Bushen in attenuating OP was investigated through network pharmacology and experimental verification, and we found that Bazi Bushen could significantly orchestrate bone homeostasis and attenuate the progression of OP by stimulating PI3K‐Akt and inhibiting apoptosis. In summary, our work sheds light on the first evidence that Bazi Bushen attenuates OP by regulating PI3K‐AKT and apoptosis pathways to orchestrate bone homeostasis.

## 1 INTRODUCTION

Osteoporosis (OP) is a common systemic skeletal disease characterized by low bone mass, bone tissue degradation and bone microarchitecture disturbance, resulting in fragility of the bones and an increased risk of fractures.^1^, ^2^, ^3^ At present, the number of people suffering from OP is estimated to exceed 200 million worldwide.^4^ By considering the factors affecting bone metabolism, OP can be classified into primary OP and secondary OP.^5^ With an aging population, the medical and socioeconomic effects of OP, particularly senile OP, will increase further.^6^ Lifestyle changes, calcium supplementation, vitamin D supplementation and anti‐OP medications such as bisphosphonates are the main ways to treat OP clinically.^7^ Nonetheless, most of them may cause side effects such as mild local injection site reactions, atypical fractures of the femur and osteonecrosis of the jaw.^7^, ^8^ Consequently, research on how to treat OP and how to reduce side effects is of great clinical importance.

Traditional Chinese medicine (TCM) formulae have unique advantages in treating OP such as Qing'e Pill, Zuogui Pill, Er‐Zhi Pill, Yougui Pill and Liuwei Dihuang Pill.^9^, ^10^, ^11^, ^12^, ^13^ By ameliorating bone loss, stimulating osteoblast bone formation, inhibiting osteoclast bone resorption and orchestrating bone homeostasis, TCM formulae can prevent or treat OP.^14^ Bazi Bushen, which consists of 16 Chinese herbs, is a Chinese patented medicine approved by the National Medical Products Administration (No. B20020585) with the function of relieving fatigue and delaying aging. The whole formula can tonify kidney, replenish essence, balance yin and yang, and delay aging. Clinical studies have proved that it can improve the symptoms of patients with kidney essence deficiency, improve exercise ability and improve the sexual function of impotence patients. Bazi Bushen has been demonstrated to improve epigenetic aging, intestinal homeostasis, skin senescence, lipid metabolism, atherogenesis, aging‐related hypogonadism and aging‐associated cognitive impairments.^15^, ^16^, ^17^, ^18^, ^19^ Several studies have also proven that Bazi Bushen could attenuate OP, such as bradykinesia and skeletal decline. Nonetheless, the pharmacological mechanisms of Bazi Bushen in attenuating OP remain predominantly unclear.

**FIGURE 1:**
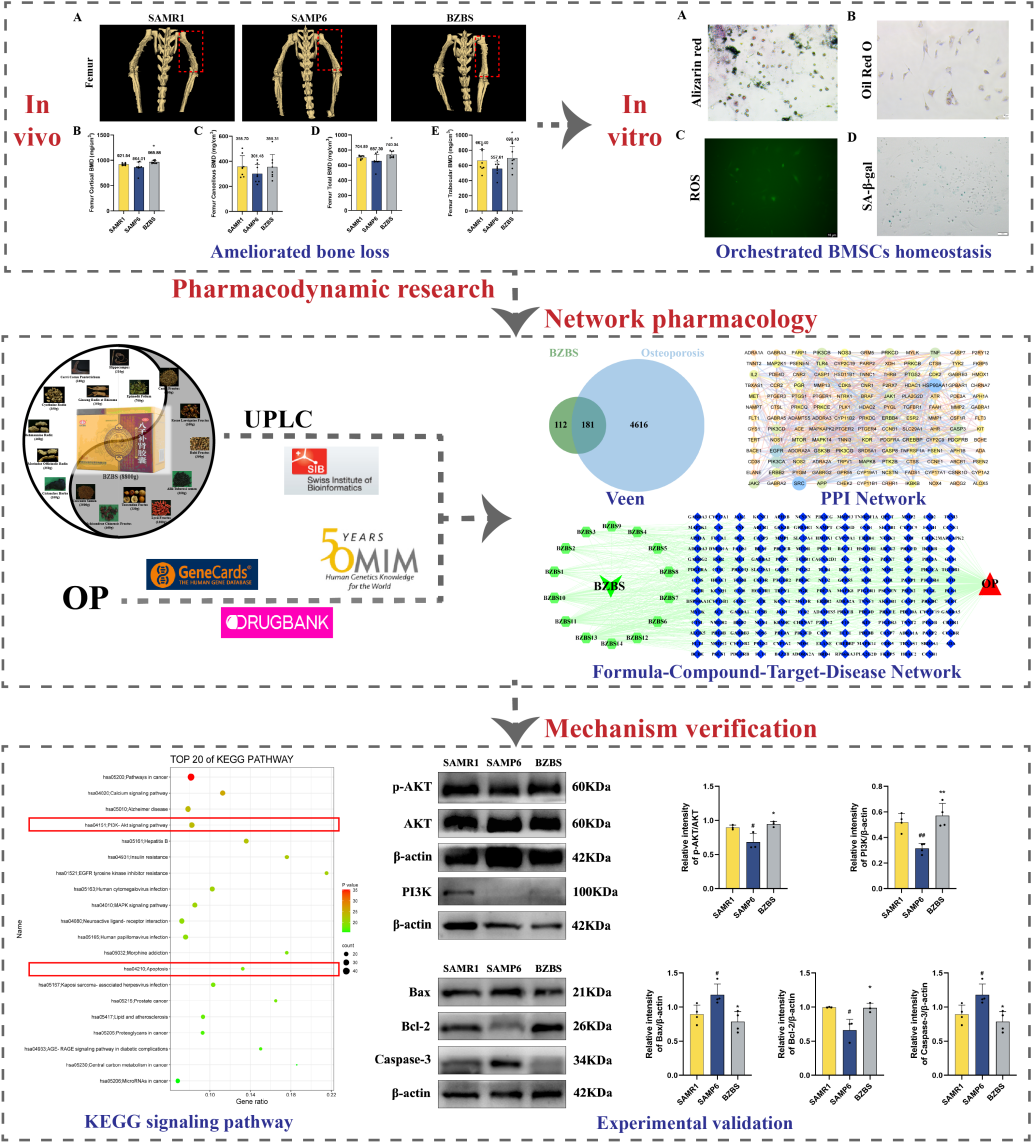
Flowchart of the pharmacological mechanism of Bazi Bushen in attenuating OP.

In this study, we verified that Bazi Bushen can improve bone homeostasis and OP in SAMP6 mice. We first investigated the pharmacodynamic research of Bazi Bushen in attenuating OP in animal and cell experiments, then explored the pharmacological mechanisms via network pharmacological predictions, and finally validated the predicted findings. The chemical compounds of Bazi Bushen were retrieved from the previous ultra‐performance liquid chromatography (UPLC) analysis.^18^, ^19^ The active targets in Bazi Bushen and their mechanism of action in attenuating OP were analysed by using network pharmacology. The core targets and pathways were selected to further verify the potential mechanisms. Our study first explored the pharmacological mechanism of Bazi Bushen in attenuating OP by utilizing animal experiments, cell experiments and network pharmacology, and provided scientific support for further clinical application. The idea of the overall study was shown in Figure 1.

## 2 MATERIALS AND METHODS

### 2.1 Chemicals and reagents

**FIGURE 2:**
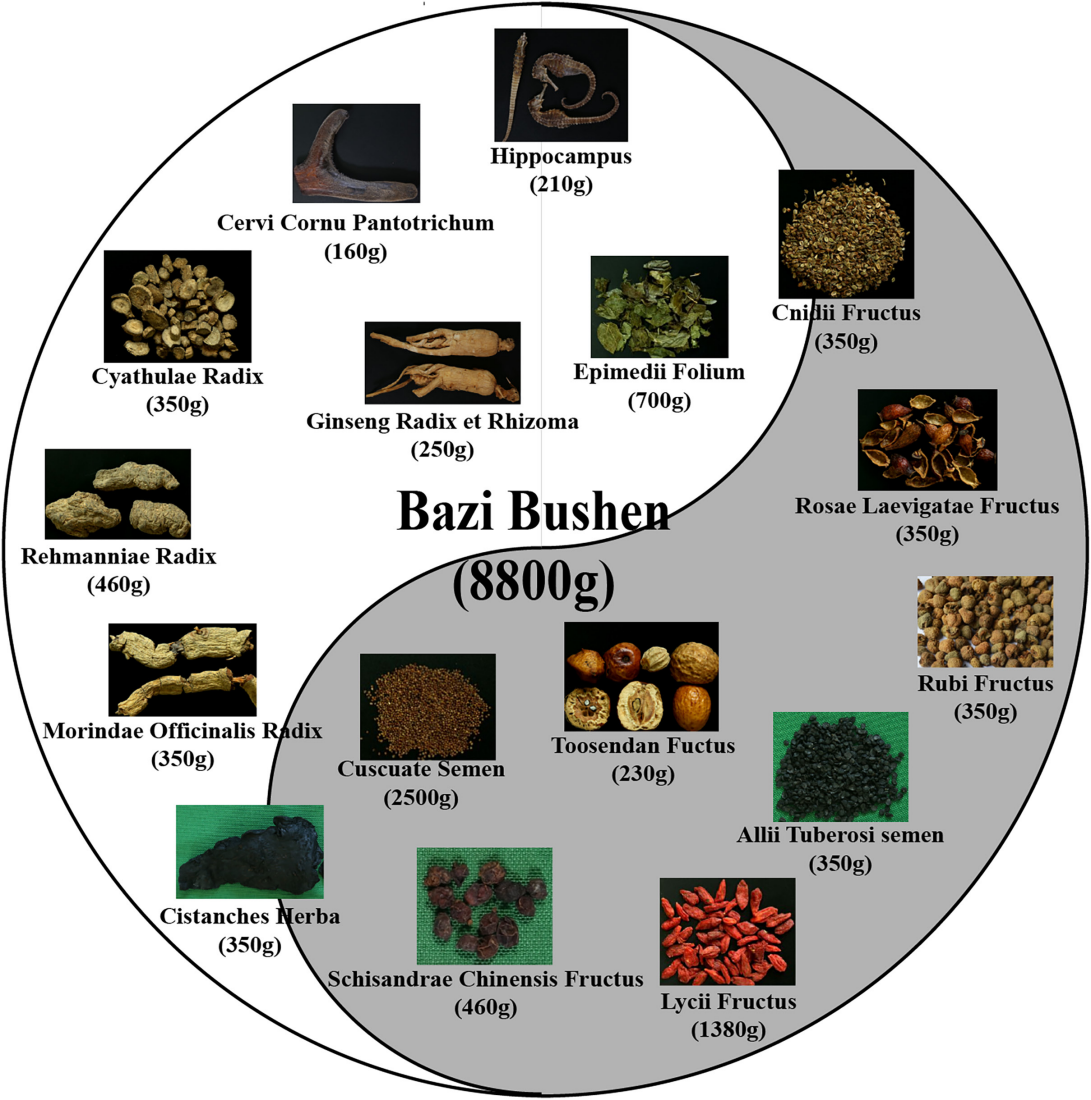
The sources and ratios of herbs in Bazi Bushen.

Bazi Bushen capsules were provided by Shijiazhuang Yiling Pharmaceutical Co., Ltd. (Lot: A2102001, Shijiazhuang, China), which was approved by the National Medical Products Administration (No. B20020585). As previously described, the preparation of Bazi Bushen has been described in sufficient detail.^18^, ^19^ The sources and ratios of herbs in Bazi Bushen were shown in Figure 2.

### 2.2 Animals and treatments

Ten male SAMR1 mice and twenty male SAMP6 mice (25–35 g) were purchased from the Department of Laboratory Animal Science of Peking University (SCXK (JING) 2016‐0010). The mice were acclimated at 24.5 ± 0.5°C under a 12 h light–dark cycle with water and food ad libitum. All animal experimental procedures were performed by the National Institutes of Health Guidelines for Laboratory Animals (NIH Publications No. 8023, revised 1978) and approved by the Ethics Committee for the Welfare of Experimental Animals of Tianjin University of Traditional Chinese Medicine under approval number TCM‐LAEC2021218. After 7 days of acclimation, the SAMR1 mice were assigned to the SAMR1 group, and the SAMP6 mice were randomly allocated to two groups (*n* = 10): SAMP6 and BZBS. The dosage of Bazi Bushen capsules (2 g/kg/day) was based on the team's previous research data and literature reports.^15^, ^16^, ^17^, ^18^, ^19^ The SAMR1 group and SAMP6 group were orally administrated with sterile saline. The mice in the BZBS group were orally administrated with 2 g/kg/day of Bazi Bushen for 9 weeks. During the experiments, the body weight and skin appearance of the mice were recorded.

### 2.3 Micro‐CT imaging

The femurs and lumbar vertebra were scanned with a small laboratory animal CT system (LaTheta LCT‐200; Aloka, Tokyo, Japan) at 40 kV and 0.25 mA for measurements placed in the 48‐mm specimen holder.^20^ Starting from the distal femur metaphysis and lumbar vertebra, 20 slices with an isotropic resolution of 48 μm were collected. The morphometric bone parameters included bone mineral density (BMD), bone mineral content (BMC), trabecular number (Tb.N), trabecular thickness (Tb.Th), trabecular separation (Tb.SP) and bone volume fraction (BV/TV). Using 3D Slicer software (https://www.slicer.org/) based on CT images, a 3D reconstruction of the femoral and lumbar scan results was performed to observe structural changes in the bone.

### 2.4 Micro‐CT analysis

The BMD and BMC were obtained automatically using LaTheta LCT‐200. The Tb.N, Tb.Th, Tb.SP and BV/TV were obtained automatically using NRecon v1.6 and CTAn v1.15 software as previously described.^20^, ^21^

### 2.5 Measurement of serum calcium and phosphorus content

According to the assay kits' instructions, the content of serum calcium and phosphorus was measured using commercial assay kits (Cat#: C006‐1‐1, C004‐2‐1; Nanjing JianCheng Bioengineering Institute, China).

### 2.6 Cell isolation and culture

To preliminarily investigate the pharmacodynamic research of Bazi Bushen in attenuating OP in cell experiments, the BMSCs from the three groups of mice were isolated and cultured according to previously reported protocols.^22^, ^23^ After the mice were sacrificed from the above groups, they were immersed in 75% medical alcohol and sterilized for 5 min. The sterilized mice were removed, and the residual alcohol was wiped off as much as possible, and transferred to an ultraclean table. The femur was removed with sterilized forceps and scissors and immediately placed in a Petri dish poured with D‐Hanks solution. The bone marrow was rinsed in the femur and tibia with D‐Hanks solution containing 10% penicillin–streptomycin. After filtration through a 70 μm filter, the filtrate was centrifuged at 300 g for 5 min. Then, the cells were collected and cultured with Dulbecco's modified Eagle's medium (DMEM, Cat#: 8121295, Gibco) containing 15% fetal bovine serum (FBS, Cat#: FBSAD‐01011‐500, Cyagen) and penicillin–streptomycin (Cat#: 15140‐112, Gibco). The cells were incubated at 37°C under 5% CO_2_. The medium was first changed after 48 h, followed by fresh medium every 2–3 days. Second‐generation BMSCs were used for the experiments. And mesenchymal stem cell (MSC) surface marker detection kit (Cat#: MUXMX‐09011, Cyagen) was used to detect BMSC surface markers.

### 2.7 Osteogenic differentiation

The osteogenic differentiation ability was tested according to previously published protocols.^24^ Second‐generation BMSCs were cultured in a 6‐well plate (2 × 10^4^ cells per well). When the cells were 60%–70% confluent, the medium was removed and 2 mL of osteogenic differentiation medium (Cat#: MUBMX‐90021, Cyagen) was added. The osteogenic differentiation medium was completely replaced every 2–3 days. After 2–4 weeks of induction, the cells were stained with alizarin red according to their morphological changes and growth. After osteogenic differentiation, the cells were washed with D‐Hanks solution, fixed with 4% paraformaldehyde for 30 min, washed with D‐Hanks solution, dyed with alizarin red dye for 3–5 min, and washed with D‐Hanks solution. The effect of osteogenic staining was observed under the microscope.

### 2.8 Adipogenic differentiation

The adipogenic differentiation ability was tested according to previously published protocols.^24^ Second‐generation BMSCs were cultured in a 6‐well plate (2 × 10^4^ cells per well). When the cells were 60%–70% confluent, the medium was removed and 2 mL of adipogenic differentiation medium (Cat#: MUBMX‐90031, Cyagen) was added. The adipogenic differentiation medium was completely replaced every 2–3 days. After 2–4 weeks of induction, the cells were stained with Oil Red O according to their morphological changes and growth. After adipogenic differentiation, they were washed with D‐Hanks solution, fixed with 4% paraformaldehyde for 30 min, washed with D‐Hanks solution, dyed with Oil Red O for 30 min, and washed with D‐Hanks solution. The effect of osteoblast staining was observed under the microscope.

### 2.9 Cellular ROS staining

Cellular ROS levels were tested according to previously published protocols.^25^ Second‐generation BMSCs were cultured in a 6‐well plate (2 × 10^5^ cells per well). Using the ROS reactive oxygen species detection kit (Cat#: R6033, Bioscience), the ROS probe was prepared, and the DCFH‐DA was diluted with serum‐free medium (1:1000). The culture medium was removed, diluted DCFHDA working solution was added, and the cells were incubated at 37°C in the dark for 30 min–4 h. Then we rinsed with serum‐free medium for 1–2 times to completely remove the DCFH‐DA that did not enter the cell. The fluorescence changes were observed directly under the fluorescence microscope.

### 2.10 Cellular SA‐β‐gal staining

Cellular aging‐related β‐galactosidase (SA‐β‐gal) staining was tested according to previously published protocols.^25^ Second‐generation BMSCs were cultured in a 6‐well plate (2 × 10^5^ cells per well). When the cells were 70%–80% confluent, the culture medium was removed, and the cells were fixed for 10 min by adding a fixation solution. The stationary solution was removed, and the cells were rinsed with PBS solution three times. The PBS solution was removed, and SA‐β‐gal staining solution (Cat#: RG0039, Beyotime) was added. Then, the cells were incubated at 37°C for 20 min–2 h or longer until some cells turned blue. The dyeing solution was removed, an appropriate amount of the PBS solution was added, and the dyeing situation was observed under the microscope.

### 2.11 Screening of active targets of Bazi Bushen and potential targets of OP

According to previous studies, the active compounds of Bazi Bushen were obtained from UPLC analysis (Table S1).^18^, ^19^ The canonical smiles of the active compounds were acquired from the PubChem database (https://pubchem.ncbi.nlm.nih.gov/). To identify the active targets of the active compounds, the canonical smiles were entered into the Swiss Target Prediction database (http://www.swisstargetprediction.ch/). Using the UniProt database (https://www.uniprot.org/), the target names were standardized. The GeneCards (https://www.Genecards.org/), OMIM (http://www.omim.org/) and Drugbank (https://go.drugbank.com/) databases were used to screen the potential targets of OP.

### 2.12 Construction of the PPI network and screening therapeutic targets

By comparing the active targets of Bazi Bushen with the potential targets of OP, therapeutic targets were screened. To construct the protein–protein interaction (PPI) network, the therapeutic targets were imported into the String Version 11.5 platform (https://string‐db.org/). High confidence intervals with scores >0.7 were selected from String and the core targets were screened out according to the degree of relevance.

### 2.13 Construction of the formula‐compound‐target‐disease network

To construct the formula‐component‐target‐disease network, the formula, compounds, therapeutic targets, and disease were imported into Cytoscape 3.8.0 software.

### 2.14 Analysis of KEGG pathway and GO function enrichment

To further investigate the role of Bazi Bushen, an overall analysis was executed utilizing the Metascape platform (http://metascape.org/gp/index.html#/main/step1) by inputting the therapeutic targets, to analyse the Kyoto Encyclopedia of Genes and Genomes (KEGG) signalling pathway and Gene Ontology (GO) function enrichment. The top 20 items of KEGG analysis and GO function enrichment were mapped as bubble plots by using the bioinformatics online website (http://www.bioinformatics.com.cn/).

### 2.15 Validation of molecular docking

The active components were chosen for preliminary molecular docking validation with the core targets. The active components and core targets were downloaded. Using config file, AutoDock vina1.1.2 software was used to calculate the molecular docking binding energy. The pdbqt format file was converted into a pdb format file in the Open Babel GUL software, and visual analysis was carried out by the PLIP web.

### 2.16 Western blotting analysis

The femoral bones were pulverized with a mortar in liquid nitrogen before being lysed with T‐Per lysis buffer, as previously reported.^23^ Total proteins were extracted from BMSCs in each group by RIPA buffer, as previously reported.^25^ The BCA assay kit was used to measure the protein concentration. The protein samples were separated by SDS‐PAGE gels and then transferred onto PVDF membranes. The protein membranes were blocked in QuickBlock Blocking Buffer (Beyotime, China) for 15 min and incubated with a 1:1000 dilution of primary antibodies separately (RUNX2, OSX, β‐actin, p16, p21, OCT4, SOX2, NANOG, GAPDH, Bax, Bcl‐2, Caspase‐3, p‐AKT, AKT and PI3K) overnight at 4°C. Then, the membranes were probed with a 1:10,000 dilution of secondary antibody for 1 h at room temperature. Immunoblots were visualized using ECL reagents (Biosharp, China) and band densities were quantified using ImageJ analyser software. β‐actin and GAPDH were used as normalization controls, and each experiment was repeated three times. RUNX2 (20700‐1‐AP), P16‐INK4A (10883‐1‐AP), and P21 (10355‐1‐AP) antibodies were provided by Proteintech Group, Inc. (Wuhan, China). OCT4 (A7920), SOX2 (A0561), and NANOG (A3232) were provided by ABclonal Technology Co., Ltd. (Wuhan, China). Phospho‐AKT (#13038), AKT (pan) (#4685), and PI3 kinase p85 (#4257) were provided by Cell Signalling Technology (MA, United States). OSX (ab209484), Bax (ab32503), Bcl‐2 (ab59348), Caspase‐3 (ab13847), Anti‐beta Actin (ab8227), GAPDH (ab181602), Goat Anti‐Rabbit IgG H&L (Alexa Fluor® 594, ab150080), and Goat Anti‐Rabbit IgG H&L (Alexa Fluor® 488, ab150077) were provided by Abcam Co., Ltd. (Cambridge, United Kingdom).

### 2.17 Statistical analysis

GraphPad PRISM8 software was used to analyse statistical significance. Measurement information was expressed as mean ± standard deviation when it conformed to normal distribution and median (interquartile spacing) when it did not work to normal distribution. When analysing the influencing factors, one‐way ANOVA was used for between‐group comparisons when the measures conformed to a normal distribution with a chi‐square variance. If the difference was statistically significant, compare the data two by two using the Bonferroni test. The Kruskal‐Wallis *H*‐test was used to compare multiple groups when the information did not fit a normal distribution, or the variance was not homogeneous, and the Mann–Whitney *U*‐test was further used for two‐by‐two comparisons when the difference was statistically significant. *p* < 0.05 was considered statistically significant.

## 3 RESULTS

### 3.1 Bazi Bushen ameliorated femoral bone loss in SAMP6 mice

**FIGURE 3:**
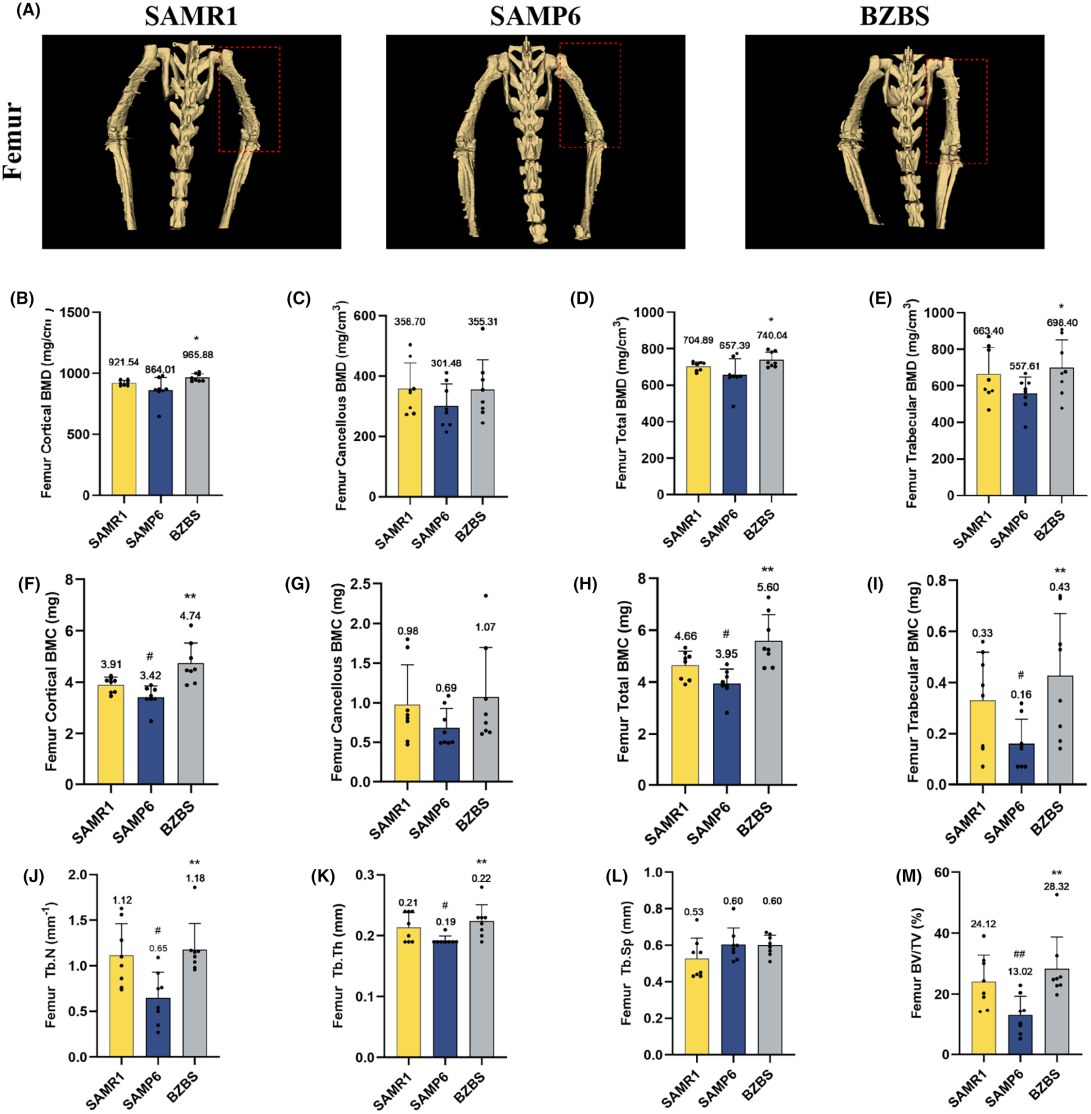
Bazi Bushen ameliorated femoral bone loss in SAMP6 mice. (A) The 3D reconstruction of femoral bone. (B) The level of femur cortical BMD. (C) The level of femur cancellous BMD. (D) The level of femur total BMD. (E) The level of femur trabecular BMD. (F) The level of femur cortical BMC. (G) The level of femur cancellous BMC. (H) The level of femur total BMC. (I) The level of femur trabecular BMC. (J–M) The level of femur Tb.N, Tb.Th, Tb.Sp and BV/TV. ^##^ *p* < 0.01, ^#^ *p* < 0.05 versus the SAMR1 group; **p < 0.01, *p < 0.05 versus the SAMP6 group (*n* = 8).

BMD and BMC were assessed to calculate the change in the inorganic mineral content of the femoral bone.^26^ The micro‐CT study revealed that when compared with SAMR1 mice, the 3D reconstruction of the femoral scan was destroyed and the femoral bone mass was markedly decreased in SAMP6 mice, whereas Bazi Bushen intervention effectively recovered the 3D reconstruction and femoral bone mass (Figure 3). Furthermore, the results of the femoral bone mass analysis revealed that Bazi Bushen improved the decline in femur cortical BMD and BMC, cancellous BMD and BMC, total BMD and BMC, and trabecular BMD and BMC in SAMP6 mice (Figure 3B–I). In parallel, compared to the SAMP6 group, we observed an increase in the femur Tb.N, Tb.Th and BV/TV in the BZBS group (Figure 3J,K,M). The femur Tb.Sp, however, exhibited no obvious restorative effect (Figure 3L). These findings indicated that Bazi Bushen enhanced the femoral BMD, BMC, Tb.N, Tb.Th and BV/TV, which can ameliorate femoral bone loss.

### 3.2 Bazi Bushen ameliorated the L1‐L4 vertebra loss in SAMP6 mice

**FIGURE 4:**
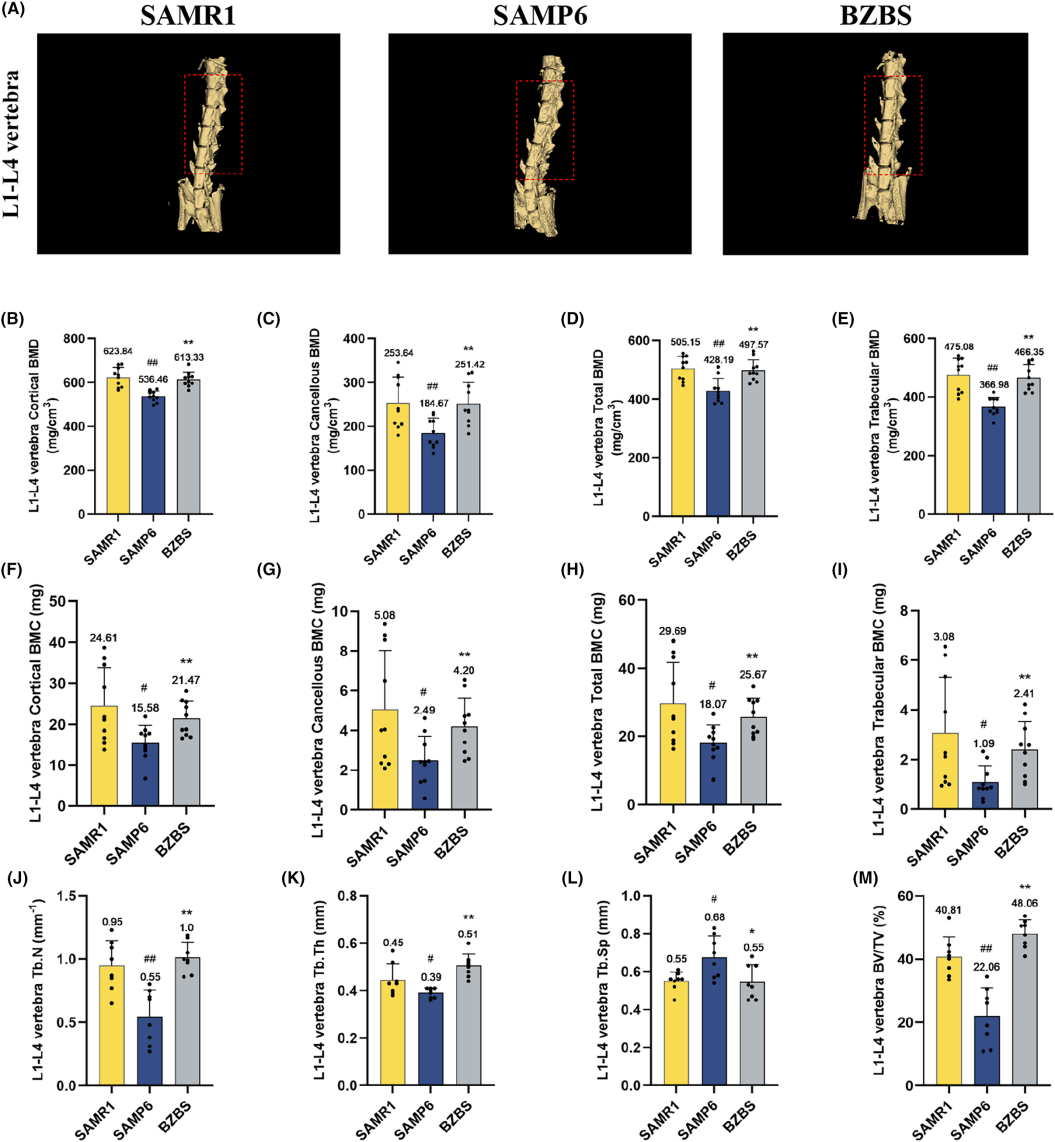
Bazi Bushen ameliorated L1‐L4 vertebra loss in SAMP6 mice. (A) The 3D reconstruction of L1‐L4 vertebra. (B) The level of L1‐L4 vertebra cortical BMD. (C) The level of L1‐L4 vertebra cancellous BMD. (D) The level of L1‐L4 vertebra total BMD. (E) The level of L1‐L4 vertebra trabecular BMD. (F) The level of L1‐L4 vertebra cortical BMC. (G) The level of L1‐L4 vertebra cancellous BMC. (H) The level of L1–L4 vertebra total BMC. (I) The level of L1‐L4 vertebra trabecular BMC. (J–M) The level of L1‐L4 vertebra Tb.N, Tb.Th, Tb.Sp and BV/TV. ^##^ *p* < 0.01, ^#^ *p* < 0.05 versus the SAMR1 group; **p < 0.01, *p < 0.05 versus the SAMP6 group (*n* = 8).

At the same time, the changes in the inorganic mineral content in the L1‐L4 vertebrae were also measured. As intended, the changes in the L1‐L4 vertebra were consistent with the femoral bone. Micro‐CT study revealed that, when compared with SAMR1 mice, the L1‐L4 vertebral mass of SAMP6 mice was markedly decreased, whereas Bazi Bushen intervention effectively recovered the L1‐L4 vertebra mass (Figure 4). Compared with the SAMP6 group, we observed an increase in the L1‐L4 vertebra BMD, BMC, Tb.N, Tb.Th and BV/TV in the BZBS group. All these results demonstrated that Bazi Bushen successfully ameliorated bone loss and the progression of OP.

### 3.3 Bazi Bushen improved the expression of osteogenic proteins in bone

**FIGURE 5:**
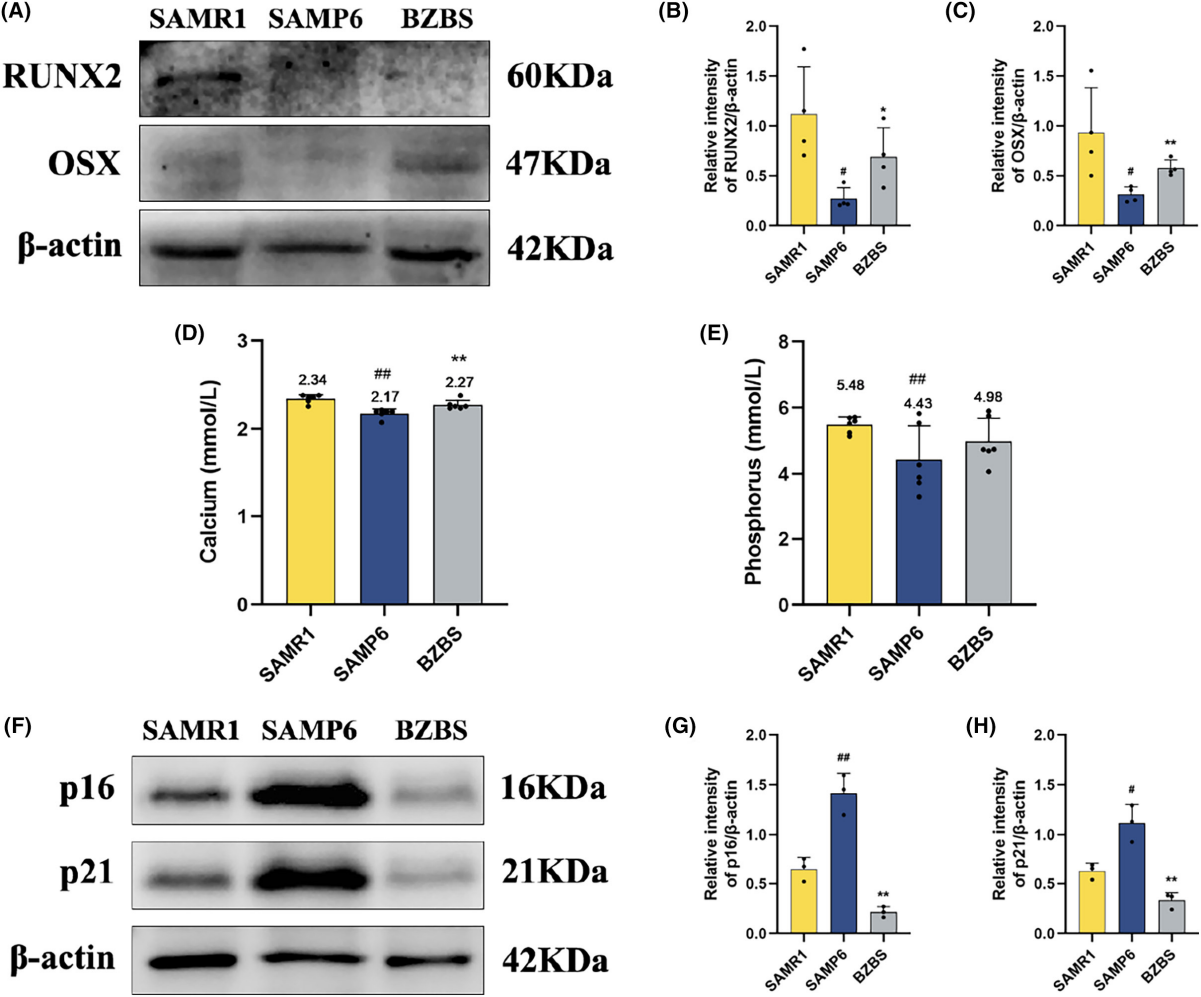
Bazi Bushen regulated key parameters of increasing OP. (A) Western blot analysis of RUNX2, OSX, and β‐Actin in bone. (B) Quantification of band intensities of RUNX2 relative to β‐Actin in bone. (C) Quantification of band intensities of OSX relative to β‐Actin in bone. (D) The content of calcium in the serum. (E) The content of phosphorus in the serum. (F) Western blot analysis of p16, p21 and β‐Actin in bone. (G) Quantification of band intensities of p16 relative to β‐Actin in bone. (H) Quantification of band intensities of p21 relative to β‐Actin in bone. ^##^ *p* < 0.01, ^#^ *p* < 0.05 versus the SAMR1 group; **p < 0.01, *p < 0.05 versus the SAMP6 group (*n* = 3–6).

To further investigate whether Bazi Bushen can affect osteogenic differentiation ability, we measured the expression levels of osteogenic differentiation markers, including RUNX2 and OSX. The results revealed that the expression of the osteogenic proteins RUNX2 and OSX in the SAMP6 group markedly decreased compared to that in SAMR1 group. Bazi Bushen treatment markedly increased the expression of RUNX2 and OSX (Figure 5A–C). These findings suggested that Bazi Bushen improved the expression of RUNX2 and OSX, which affected osteogenic differentiation ability and attenuated OP.

### 3.4 Bazi Bushen regulated serum calcium and phosphorus content in SAMP6 mice

Low levels of calcium and phosphorus, aging, and oestrogen deficiency are key parameters of increasing OP.^26^, ^27^ Therefore, we investigated the effect of Bazi Bushen on the levels of serum calcium and phosphorus. The levels of serum calcium and phosphorus were lower in the SAMP6 group than in the SAMR1 group (Figure 5D,E). Bazi Bushen treatment slightly upregulated the levels of serum calcium and phosphorus. These findings suggest that Bazi Bushen may attenuate OP by regulating the levels of serum calcium and phosphorus.

### 3.5 Bazi Bushen improved the expression of aging‐related proteins in bone

OP is frequently linked to ailments caused by aging. During the development of physiological aging and aging‐related diseases, the expression of p16 and p21 is gradually increased.^28^, ^29^ To confirm Bazi Bushen's effect on bone aging, we measured the expression of the aging‐related proteins p16 and p21 in bone. The results revealed that the expression of p16 and p21 in the SAMP6 group markedly increased compared with that in SAMR1 group. Bazi Bushen treatment markedly decreased the expression of p16 and p21 (Figure 5F–H). These findings suggested that Bazi Bushen can improve the expression of aging‐related proteins, which attenuated bone aging and OP.

### 3.6 Cell culture and identification

To preliminarily investigate the pharmacodynamic research of Bazi Bushen in attenuating OP in cell experiments, we successfully isolated BMSCs from the three groups of mice and cultured them in a culture flask. The cells were circular, of various sizes, and suspended in the culture medium according to the protocols.^22^, ^23^, ^30^ After 48 h of culture, the medium was completely changed, and some of the suspended cells began to adhere to the wall and grew in a short fusiform and polygonal shape (Figure S1A). After 3 to 4 days of culture, cell colonies arranged radially were observed, and the cells were mainly spindle‐shaped (Figure S1B). After 6 to 7 days of culture, the cells grew in colonies, with 80% to 90% confluence, swirled, and arranged in the same direction (Figure S1C). After 9 to 10 days of culture, the cells were tightly packed and gradually fused into sheets (Figure S1D). Second‐generation cells with good growth status were collected, and surface markers of BMSCs were detected by flow cytometry. The results showed that the second‐generation cells expressed CD44 with a positive rate of 66.83%. However, the expression of CD117 was negative, with a negative rate of 1.67% (Figure S2). These results indicated that the cultured cells were BMSCs. Then, we used a CCK‐8 assay to detect cell viability in the SAMR1, SAMP6, and BZBS groups after 24 h of adherence and after 96 h of culture. The results showed that at 24 h, the cells in the three groups were adherent and growing with the same level of cell viability (Figure S3). However, with the extension of culture time, the cell viability of the SAMR1 and BZBS groups tended to increase compared to that of the SAMP6 group, although the difference was not significant.

### 3.7 Bazi Bushen improved the osteogenic differentiation and inhibited adipogenic differentiation in BMSCs

**FIGURE 6:**
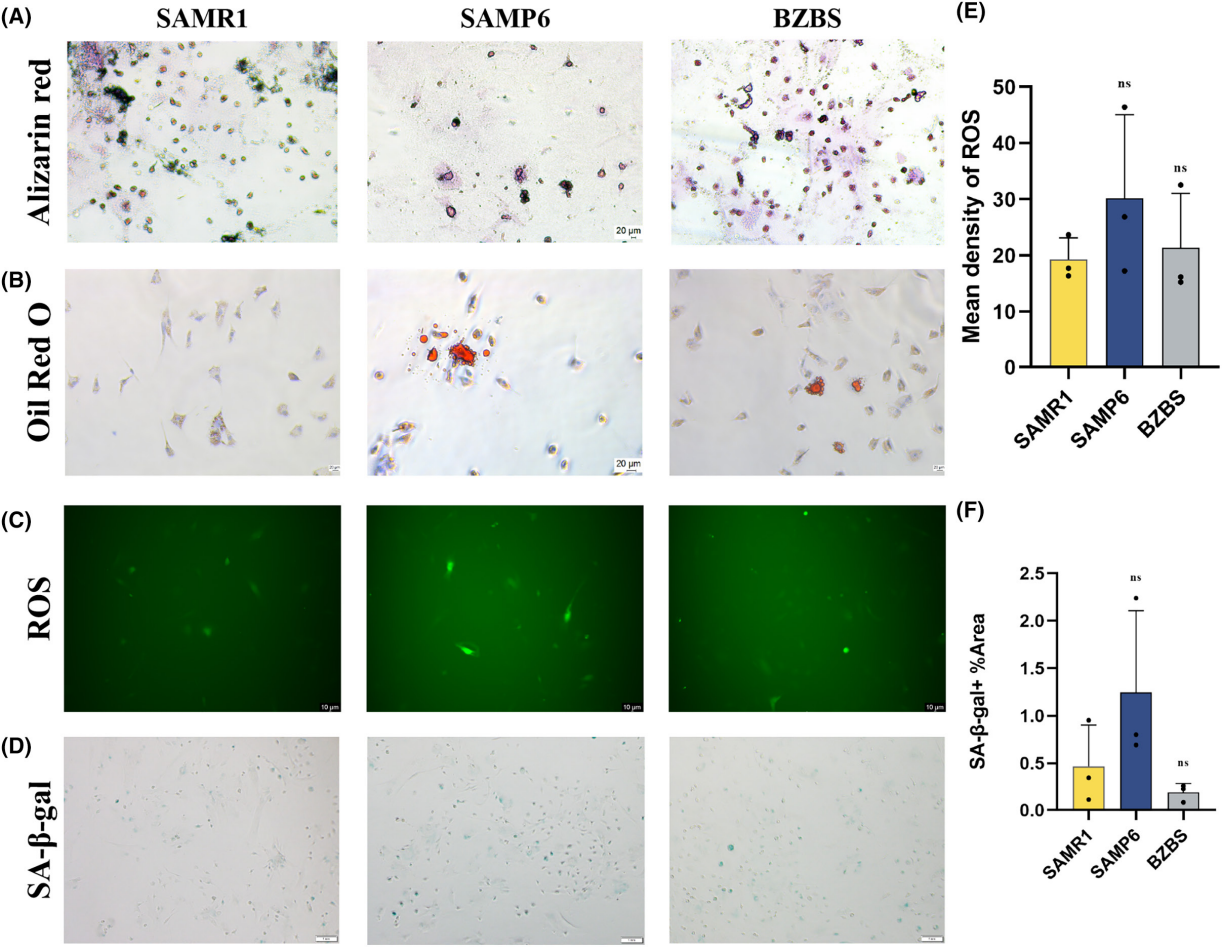
Bazi Bushen improved bone homeostasis in BMSCs. (A) Representative images of alizarin red staining in BMSCs. (B) Representative images of oil red O staining in BMSCs. (C) Representative images of ROS‐induced fluorescence in BMSCs. (D) Representative images of SA‐β‐gal staining in BMSCs. (E) Quantitative image analysis of ROS based on the mean density. (F) Quantitative image analysis of the area of SA‐β‐gal‐positive cells. ^##^ *p* < 0.01, ^#^ *p* < 0.05 versus the SAMR1 group; **p < 0.01, *p < 0.05 versus the SAMP6 group (*n* = 3).

Second‐generation cells with good growth status were collected and operated according to the protocols of osteogenic and adipogenic differentiation.^24^, ^31^ BMSCs from each group were induced with osteogenic differentiation medium for 21 days and stained with an alizarin red staining solution. Compared to the SAMR1 group, the number of red mineralized nodules was significantly reduced in the SAMP6 group (Figure 6A). However, compared to the SAMP6 group, the number of red mineralized nodules was improved in the BZBS group.

BMSCs from each group were induced by adipogenic differentiation medium for 26 days and then stained with Oil Red O staining solution. The results are shown in Figure 6B. Compared to the SAMR1 group, the number of lipid droplets stained was significantly increased in the SAMP6 group. However, the number of lipid droplets stained in the BZBS group was decreased compared to that in the SAMP6 group. These results indicated that BZBS increased the osteogenic differentiation potential of BMSCs and decreased the adipogenic differentiation potential of BMSCs. Bazi Bushen was able to improve the balance of osteogenic and adipogenic differentiation in BMSCs.

### 3.8 Bazi Bushen improved ROS levels and SA‐β‐gal staining in BMSCs

The ROS levels of cells in the SAMR1, SAMP6 and BZBS groups were detected using the fluorescent probe DCFH‐DA.^25^, ^32^ Compared to that of the SAMR1 group, the fluorescence signal intensity of the SAMP6 group was significantly increased. Compared to that in the SAMP6 group, the fluorescence signal intensity in the BZBS group was enhanced (Figure 6C). These results indicated that the ROS levels of BMSCs in the BZBS group decreased, suggesting that Bazi Bushen could improve the ROS levels in BMSCs.

In situ β‐galactosidase staining was used to detect cell senescence in the SAMR1, SAMP6 and BZBS groups.^25^, ^32^ Compared to that in the SAMR1 group, the number of SA‐β‐gal positive senescent cells in the SAMP6 group was significantly increased. However, compared to that in the SAMP6 group, the number of SA‐β‐gal positive senescent cells was improved in the BZBS group (Figure 6D). This indicated that the BZBS group BMSC senescence levels were reduced, which indicated that Bazi Bushen could alleviate BMSC senescence in the SAMP6 mice.

### 3.9 Bazi Bushen increased the expression of stemness genes in BMSCs

**FIGURE 7:**
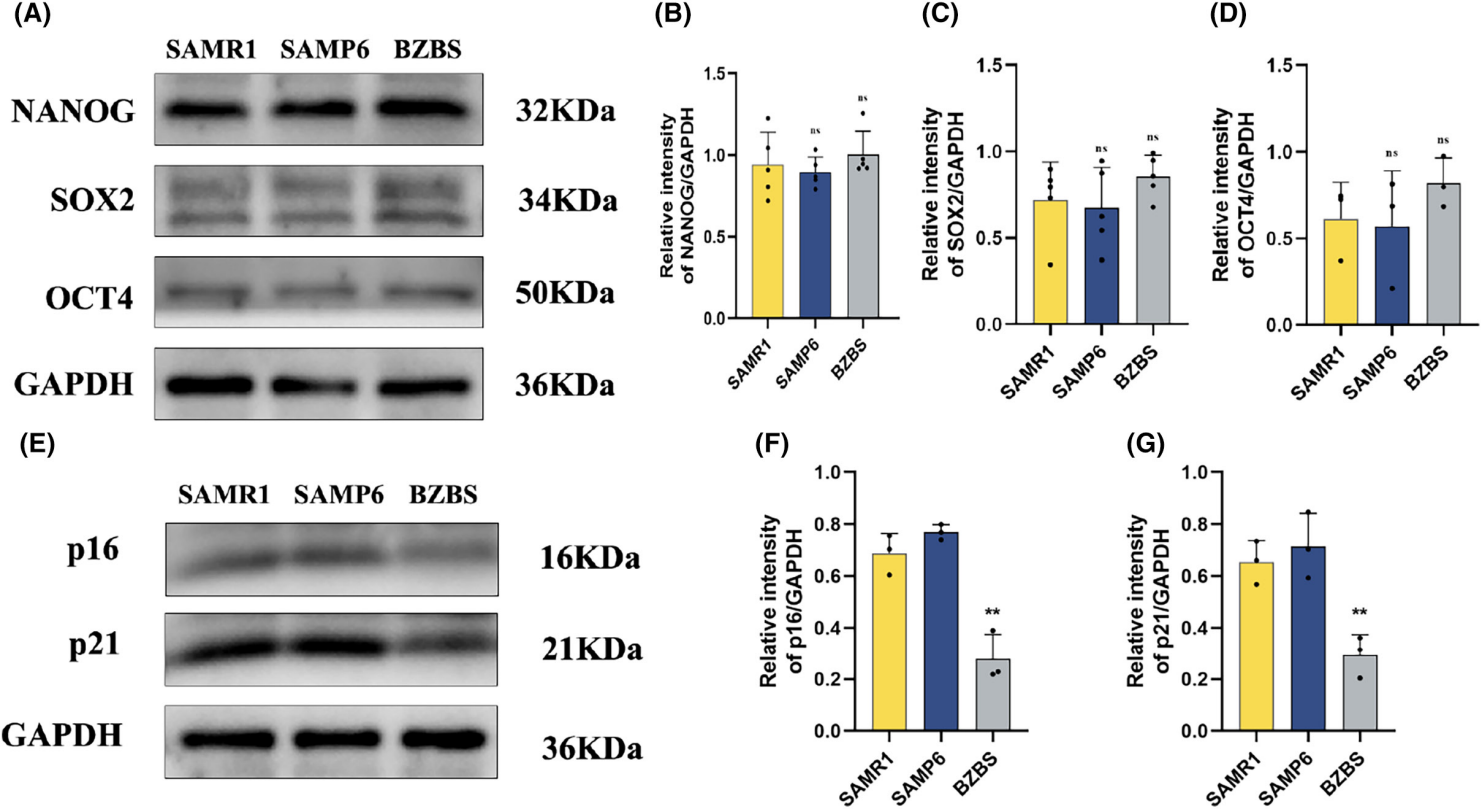
Bazi Bushen improved the expression of stemness biomarkers and aging‐related proteins in BMSCs. (A) Western blot analysis of NANOG, SOX2, OCT4 and GAPDH in BMSCs. (B) Quantification of band intensities of NANOG relative to GAPDH in BMSCs. (C) Quantification of band intensities of SOX2 relative to GAPDH in BMSCs. (D) Quantification of band intensities of OCT4 relative to GAPDH in BMSCs. (E) Western blot analysis of p16, p21 and GAPDH in BMSCs. (F) Quantification of band intensities of p16 relative to GAPDH in BMSCs. (G) Quantification of band intensities of p21 relative to GAPDH in BMSCs. ^##^ *p* < 0.01, ^#^ *p* < 0.05 versus the SAMR1 group; **p < 0.01, *p < 0.05 versus the SAMP6 group (*n* = 3–4).

To further investigate whether Bazi Bushen could affect the stemness genes in BMSCs, we examined the expression of the stemness biomarkers NANOG, SOX2 and OCT4 in BMSCs.^33^ The results showed that the expression of stemness biomarkers in BMSCs was decreased in the SAMP6 group compared to the SAMR1 group. Bazi Bushen treatment increased the protein expression of the stemness genes in BMSCs (Figure 7A–D). These results indicated that Bazi Bushen could improve the expression of the stemness biomarkers NANOG, SOX2 and OCT4 proteins, thereby affecting the stemness of BMSCs in SAMP6 mice.

### 3.10 Bazi Bushen improved the expression of aging‐related proteins in BMSCs

In vivo experiments have confirmed that Bazi Bushen can improve bone aging by acting on the expression of aging‐related proteins in bone tissue. To investigate whether Bazi Bushen may affect BMSC senescence, we detected the expression of the aging‐related proteins p16 and p21 in BMSCs from each group.^34^ The results showed that the BMSCs in the SAMP6 group had higher expression of the proteins p16 and p21 than those in the SAMR1 group. Bazi Bushen treatment reduced the protein expression of p16 and p21 in BMSCs (Figure 7E–G). It has been suggested that Bazi Bushen can not only improve the expression of aging‐related proteins in bone tissue, but also improve the expression of aging‐related proteins in BMSCs, thus improving bone aging and attenuating OP.

### 3.11 Active targets of Bazi Bushen and potential targets of OP

**FIGURE 8:**
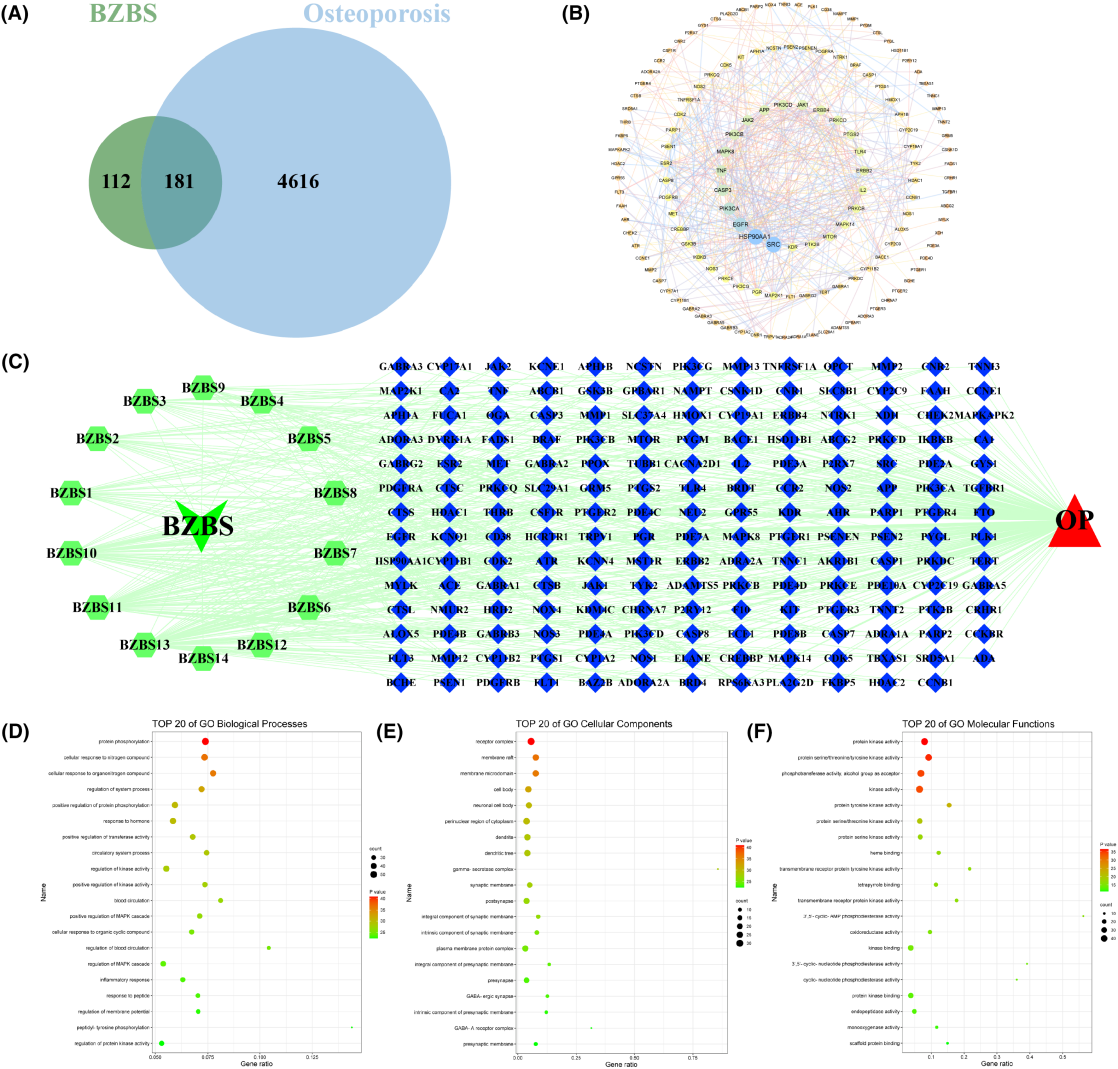
The PPI network, formula‐compound‐target‐disease network, and GO function enrichment construction. (A) The intersection targets of active targets from Bazi Bushen and OP potential targets. (B) The PPI network of 181 therapeutic targets of Bazi Bushen against OP. (C) The network of formula‐compound‐target‐disease includes 14 active components and 181 target genes. (D) The top 20 of GO function enrichment in Biological Process. (E) The top 20 of GO function enrichment in Cellular Component. (F) The top 20 of GO function enrichment in Molecular Function.

A total of 293 active targets from 14 compounds derived from Bazi Bushen were retrieved from the Swiss Target Prediction database according to the screening criteria of probability >0. A total of 4797 potential targets were retrieved from three databases: Genecards, OMIM and DrugBank. A total of 293 active targets of Bazi Bushen were intersected with 4797 potential targets of OP to find 181 therapeutic targets (Figure 8A).

### 3.12 Construction of the PPI network and the formula‐compound‐target‐disease network

A total of 181 therapeutic targets were imported into the STRING platform for PPI visualization (Figure 8B), and the core targets were screened out. The Bazi Bushen, 14 compounds, 181 therapeutic targets and OP were imported into Cytoscape 3.8.0 software to construct the formula‐compound‐target‐disease network (Figure 8C).

### 3.13 Analysis of KEGG pathway and GO function enrichment

A total of 188 KEGG pathways were obtained by introducing 181 therapeutic targets into the Metascape platform for KEGG pathway enrichment analysis. The top‐ranked pathways comprised mainly cancer, calcium, PI3K‐AKT, MAPK, apoptosis, and lipid and atherosclerosis signalling pathways. The top 20 pathways are displayed, and the bubble diagram for enrichment analysis was shown in Figure 8C. Furthermore, we discovered that the PI3K‐AKT and apoptosis pathways were crucial biological processes for Bazi Bushen in treating OP. A total of 181 therapeutic targets were also imported into the Metascape platform for GO biological process (BP), cellular component (CC), and molecular function (MF) enrichment analysis. A total of 1624 BP entries, 134 CC entries and 195 MF entries were obtained. The top 20 molecular function entries for GO BP, CC and MF were selected for enrichment analysis histograms (Figure 8D–F). The results showed that BP was mainly related to the regulation of systematic processes, positive regulation of protein phosphorylation, etc. CC mainly included the receptor complex, membrane raft, neuronal cell body, perinuclear region of cytoplasm, etc. MF mainly included protein serine/threonine kinase activity, etc.

### 3.14 Validation of molecular docking

**FIGURE 9:**
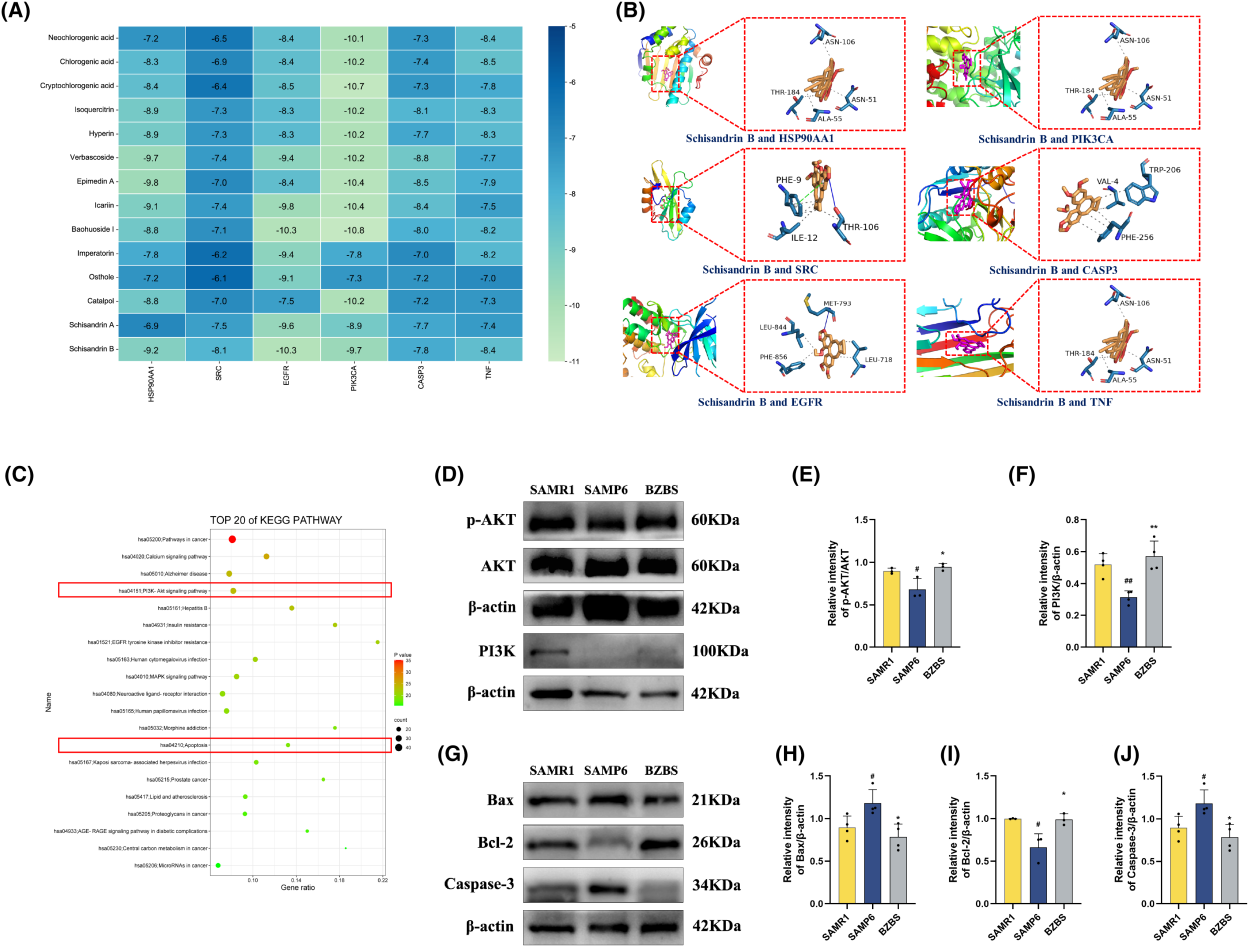
Analysis of molecular docking and KEGG pathway. (A) The molecular docking heat‐map of the active components and core targets. Affinity: Kcal/mol. (B) The molecular docking diagram. (C) The top 20 of KEGG Pathway enrichment analysis. (D) Western blot analysis of p‐AKT, AKT, PI3K, and β‐Actin in bone. (E) Quantification of band intensities of p‐AKT relative to AKT in bone. (F) Quantification of band intensities of PI3K relative to β‐Actin in bone. (G) Western blot analysis of Bax, Bcl‐2, Caspase‐3, and β‐Actin in bone. (H) Quantification of band intensities of Bax relative to β‐Actin in bone. (I) Quantification of band intensities of Bcl‐2 relative to β‐Actin in bone. (J) Quantification of band intensities of Caspase‐3 relative to β‐Actin in bone. ^##^ *p* < 0.01, ^#^ *p* < 0.05 versus the SAMR1 group; **p < 0.01, *p < 0.05 versus the SAMP6 group (*n* = 3–5).

The active compounds of Bazi Bushen were molecularly docked and combined with the core targets, including HSP90AA1(1BYQ), SRC(1O43), EGFR(5UG9), PIK3CA(7JIU), CASP3(2DKO) and TNF(5UUI). In general, the more stable the conformation of a small molecule ligand bound to a large molecule receptor, the lower the energy and the greater the likelihood of interaction, with docking binding energies <−5 kcal/mol indicating that a stable docking structure can be formed.^35^ The results showed that all 14 active compounds of Bazi Bushen were well bound to the core targets, and the compound with the lowest binding energy to six core targets was Schisandrin B (Figure 9A,B).

### 3.15 Bazi Bushen stimulated PI3K‐AKT and inhibited apoptosis pathways of OP

Our network pharmacology study predicted that the PI3K‐AKT and apoptotic pathways were most closely associated with Bazi Bushen in treating OP. Therefore, we used western blotting to determine the levels of p‐AKT, AKT and PI3K, which are essential proteins in the PI3K‐AKT signalling pathway, in bone (Figure 9D–F). The results revealed that the levels of p‐AKT and PI3K in the SAMP6 group decreased compared with those in the SAMR1 group. After treatment with Bazi Bushen, the levels of p‐AKT and PI3K markedly increased, which indicated that the PI3K‐AKT signalling pathway was stimulated. Then, to confirm Bazi Bushen's effect on the apoptosis signalling pathway, we measured the levels of Bax, Bcl‐2, and Caspase‐3 (Figure 9G–J). As expected, the levels of Bax and Caspase‐3 substantially increased and the levels of Bcl‐2 markedly decreased in the SAMP6 group compared with the SAMR1 group, whereas Bazi Bushen intervention effectively recovered the situation. These findings indicated that Bazi Bushen may stimulate the PI3K‐AKT pathway and inhibit the apoptosis pathway to attenuate OP in SAMP6 mice.

## 4 DISCUSSION

**FIGURE 10:**
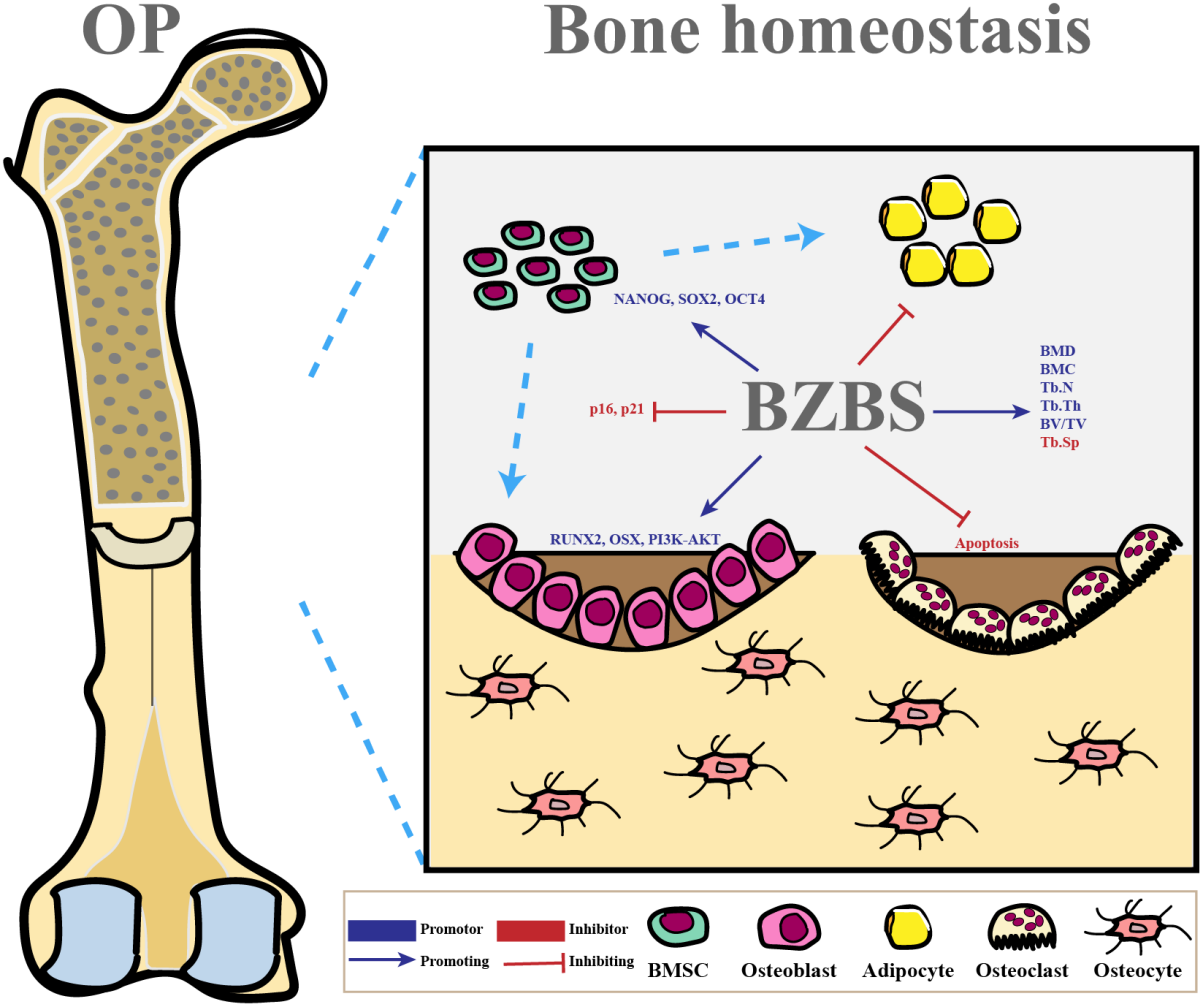
Bazi Bushen attenuates osteoporosis via orchestrating bone homeostasis.

OP is a systemic skeletal disease that reduces bone mass and increases fracture risk.^1^, ^2^ The balance between bone formation and resorption is critical for maintaining bone mass and systemic mineral homeostasis. When this balance is impaired, normal bone remodelling cannot stabilize bone mass, leading to osteopenia and OP.^36^ Studies have shown that TCM formulae can prevent or treat OP by ameliorating bone loss, stimulating osteoblast bone formation, inhibiting osteoclast bone resorption, and orchestrating bone homeostasis.^14^, ^37^ Bazi Bushen, a TCM formula, is used to relieve fatigue and delay aging. Bazi Bushen has been demonstrated to be effective in attenuating OP, but the pharmacological mechanism remains predominantly unclear. Network pharmacology can effectively uncover and validate the core pathways and pharmacological mechanisms.^38^ In the present study, we first investigated the pharmacodynamic research of Bazi Bushen in attenuating OP in animal and cell experiments, then explored the pharmacological mechanisms via network pharmacological predictions, and finally validated the predicted findings. Bazi Bushen attenuates OP in SAMP6 mice by stimulating PI3K‐AKT and inhibiting apoptosis pathway to orchestrate bone homeostasis (Figure 10).

SAMP6 mice have been used as a model of senile OP and exhibited obesity and lower bone mass compared to SAMR1 mice.^39^, ^40^ To confirm the core pathway of Bazi Bushen in attenuating OP, SAMP6 mice were employed and provided Bazi Bushen intragastrically for 9 weeks. In our experiments, we found the same phenomenon: mice in the SAMP6 group exhibited lower bone mass than those in the SAMR1 group. After treatment with Bazi Bushen, the low bone mass was reversed. There are no symptoms of bone loss, and measurements of BMD and BMC have long been recognized as crucial diagnostic indicators of OP.^26^ Bazi Bushen treatment increased cortical BMD and BMC, cancellous BMD and BMC, total BMD and BMC, trabecular BMD and BMC, Tb.N, Tb.Th and BV/TV in the femoral bone and L1‐L4 vertebra. Notably, the active compounds in Bazi Bushen, such as neochlorogenic acid, chlorogenic acid, isoquercitrin, epimedin A, icariin, baohuoside I, imperatorin, osthole and schisandrin B could prevent bone loss, inhibit the development of OP and treat bone disorders.^41^, ^42^, ^43^, ^44^, ^45^ Neochlorogenic acid has been reported to repress the TNF‐induced formation of osteoclasts and decrease erosions of bone.^42^ Baohuoside I could inhibit osteoclast differentiation and protect against bone loss following ovariectomy.^44^ Altogether, our results support the beneficial role of Bazi Bushen in the treatment of OP in SAMP6 mice.

Recently, an increasing number of studies have shown that TCM formulae can increase the expression of osteogenic markers such as RUNX2 and OSX to regulate adipogenic differentiation and attenuate OP.^46^ RUNX2, a transcription factor, regulates the expression of early osteoblast‐specific genes and keeps osteoblastic cells in an immature state.^47^ RUNX2 was reported to respond to growth factor signalling and regulate the expression of its downstream genes, including ALP, OSX, and OCN.^46^ OSX is a master gene that controls osteoblast lineage commitment and subsequent adipogenic differentiation and proliferation.^47^ Consistent with the above studies, Bazi Bushen treatment markedly increased the expression of the osteogenic proteins RUNX2 and OSX, which demonstrated that Bazi Bushen can regulate adipogenic differentiation.

Several parameters, such as low levels of calcium and phosphorus, aging and oestrogen deficiency, could increase the risk of OP.^42^, ^45^ Serum biomarkers for OP, such as serum osteocalcin, calcium, alkaline phosphatase and phosphorus, are associated with bone remodelling by osteoblasts and osteoclasts.^27^, ^48^ Combined with the measurement of BMD, the clinical applications of serum biomarkers have provided comprehensive information for the diagnosis of OP.^48^ Reports have shown that bone turnover in animals with experimental OP is related to a significant increase in urinary calcium and phosphorus, together with a decrease in serum calcium and phosphorus.^49^, ^50^ Compared with SAMR1, the urinary excretion rates of cAMP and calcium in SAMP6 were higher throughout the whole experiment, and the urinary excretion rates of hydroxyproline and phosphorus were higher after 8 weeks.^50^ In the current study, it was noticed that the serum calcium and phosphorus levels were decreased in SAMP6. Bazi Bushen treatment slightly upregulated the levels of serum calcium and phosphorus. These findings suggest that Bazi Bushen may attenuate OP by regulating the levels of serum biomarkers calcium and phosphorus.

Age‐related bone loss is largely the result of aging mechanisms that affect the quality and function of osteocytes.^51^ With aging, osteoprogenitors, osteoblasts, osteocytes and myeloid cells in the bone microenvironment become senescent. There is growing evidence that osteoporotic bone from aged mice and elderly people shows the presence of senescent cells and components of the senescence‐associated secretory phenotype. The number of senescent cells and aging‐related proteins in bone tissue increased, such as the expression of p16 and p21 in osteocytes. Moreover, several studies have shown that targeted p16 and p21 can stimulate osteoblast bone formation and inhibit osteocyte senescence and osteoclast bone resorption.^52^ Bazi Bushen treatment significantly decreased the expression of p16 and p21, which proved that Bazi Bushen can regulate bone aging and improve bone quality and function.

BMSCs are important cells with self‐proliferation and multi‐directional differentiation capabilities and can differentiate into osteoblasts, adipocytes, and chondrocytes. The balance of osteogenic and adipogenic differentiation can maintain bone homeostasis.^53^ The aging of BMSCs is mainly manifested in the significant reduction of proliferation, differentiation, and tissue repair ability. The aging and differentiation changes of BMSCs are one of the main causes of senile OP. Studies have reported that senescent BMSCs have reduced cell viability and the number of differentiations into osteoblasts, which leads to reduced bone formation. Targeting BMSC senescence can alleviate bone loss in aging mice.^54^ In the process of senile OP, the decrease of osteogenic differentiation in senescent BMSCs is accompanied by an increase in adipogenic differentiation, leading to a decrease in bone mass and an increase in bone marrow adipose tissue.

To preliminarily investigate the pharmacodynamic research of Bazi Bushen in attenuating OP in cell experiments, we successfully isolated BMSCs from the three groups of mice and detected BMSC cell surface markers. In light of the SAMP6 strain mice of our experiments and because the last intervention was performed when mice were 20 weeks old, the content and purity of BMSCs in the mice were low, and the proliferative activity was reduced. This was consistent with the results of previous studies,^55^, ^56^ and the loss of the expression of cell surface markers may be correlated with the senescent‐like phenotype of BMSCs in SAMP6 mice. Bazi Bushen could improve the balance of osteogenic and adipogenic differentiation of BMSCs in SAMP6 mice. It also improved the ROS levels, BMSC senescence, the expression of stemness biomarkers NANOG, SOX2 and OCT4 proteins, and the expression of aging‐related proteins in BMSCs of SAMP6 mice. Although some cell experiments did not show significant differences, they still provided us with preliminary evidence that Bazi Bushen capsule could improve the proliferation and differentiation ability of BMSCs and improve the expression of aging‐related proteins not only in bone tissue but also in BMSCs, thereby attenuating OP and orchestrating bone homeostasis.

Based on network pharmacology analysis, 14 active compounds and 293 active targets of Bazi Bushen were retrieved, and 4797 potential targets of OP were obtained, including 181 therapeutic targets. The results showed that all 14 active compounds of Bazi Bushen were well bound to these core targets, and the compound with the lowest binding energy to these core targets was Schisandrin B. The results were consistent with previous studies, which showed that Schisandrin B was effective against OP and other osteoclast‐related diseases.^41^ Increasingly studies have revealed that the PI3K‐AKT signalling pathway is important for the regulation of OP.^14^, ^37^ The differentiation of skeletal component cells, such as osteoblasts, chondrocytes, myoblasts, and adipocytes, is significantly influenced by the PI3K‐AKT pathway. The PI3K‐AKT signalling pathway is involved in OP inhibition by promoting osteoblast proliferation, differentiation and bone formation. Previous studies have shown that TCM formulae can regulate the PI3K‐AKT pathway, stimulate osteoblast bone formation, and inhibit osteoclast bone resorption.^57^ Our pharmacological study revealed that the PI3K‐AKT pathway was most closely associated with the ability of Bazi Bushen to attenuate OP. Apoptosis is closely related to maintaining the balance between bone formation and bone resorption. On the one hand, earlier studies have suggested that increased osteoblast apoptosis may worsen the course of OP, and inhibiting the apoptosis of osteoblasts can significantly enhance the differentiation of osteoblasts.^14^, ^37^ On the other hand, osteoblast apoptosis is impacted by the stimulated PI3K‐AKT signalling pathway. In general, our findings support that Bazi Bushen can stimulate osteoblast bone formation and inhibit osteoclast bone resorption to attenuate OP by regulating the PI3K‐AKT and apoptosis pathways.

Bazi Bushen can considerably attenuate the progression of OP and orchestrate bone homeostasis by stimulating PI3K‐AKT and inhibiting apoptosis. However, there are some limitations of the present study. First, we did not evaluate the effect of Bazi Bushen on the natural aging mice. However, SAMP6 mice have been used as a model of senile OP and exhibited lower bone mass compared to SAMR1 mice. Meanwhile, treatment with Bazi Bushen can improve bone structure and attenuate OP. Second, the multi‐dose studies will be needed and the dose–response curve in the experimental setup should be further clarified and detailed. Third, only the core pathway has been verified, and further studies are needed, including exploring other mechanisms and cell experiments.

## 5 CONCLUSIONS

Based on our findings, Bazi Bushen can serve as a potential therapy for treating OP, orchestrating bone homeostasis, and attenuating the progression of OP by activating PI3K‐AKT and inhibiting apoptosis. This could expand the scope of TCM formulae in attenuating OP and provide references for clinical promotion and ideas.

## AUTHOR CONTRIBUTIONS

Zhe Xu: Conceptualization (equal); data curation (equal); formal analysis (equal); methodology (equal); resources (equal); writing—original draft (equal). Zeyu Zhang: Formal analysis (equal); methodology (equal); resources (equal). Huifang Zhou: Conceptualization (equal); formal analysis (equal); methodology (equal); project administration (equal); supervision (equal). Shan Lin: Investigation (equal); resources (equal). Boyang Gong: Investigation (equal); resources (equal). Zhaodong Li: Investigation (equal); resources (equal). Shuwu Zhao: Visualization (equal); writing—review & editing (equal). Yunlong Hou: Visualization (equal); writing—review & editing (equal). Yanfei Peng: Conceptualization (equal); formal analysis (equal); methodology (equal); project administration (equal); supervision (equal). Yuhong Bian: Conceptualization (equal); formal analysis (equal); project administration (equal); supervision (equal); writing—review & editing (equal).

## FUNDING INFORMATION

The S&T Program of Hebei (No.23379902 L), the National Key R&D Program of China (2018YFC1706500), the Strategic Research and Consulting Project of the Chinese Academy of Engineering (2022‐XY‐45), and the Tianjin Municipal Education Commission Science and Technology Plan Project (2021KJ137).

## CONFLICT OF INTEREST STATEMENT

The authors declare that the research was conducted in the absence of any commercial or financial relationships that could be construed as a potential conflict of interest.

## Supporting information


Data S1.

## Data Availability

Data can be accessed by emailing the corresponding authors.
