# Supplementary material for: Bazi Bushen attenuates osteoporosis in SAMP6 mice by regulating PI3K‐AKT and apoptosis pathways
Source: J Cell Mol Med. 2024 Oct 29;28(20):e70161. doi: 10.1111/jcmm.70161 (PMC11519748; doi:10.1111/jcmm.70161)
Supplement: Supplementary file 1 — Data S1. [file JCMM-28-e70161-s001.docx]

**Supplementary material**

**for**

**Bazi Bushen attenuates osteoporosis in SAMP6 mice by regulating PI3K-AKT and apoptosis pathways**

Zhe Xu^1,2^, Zeyu Zhang^1,2^, Huifang Zhou^1^, Shan Lin^1,2^, Boyang Gong^1,2^, Zhaodong Li^1,2^, Shuwu Zhao^1^, Yunlong Hou^3^, Yanfei Peng^1^, Yuhong Bian^1^

^1^ School of Integrative Medicine, Tianjin University of Traditional Chinese Medicine, Tianjin 301617, P.R China.

^2^ Graduate School, Tianjin University of Traditional Chinese Medicine, Tianjin 301617, P.R China.

^3^ National Key laboratory of Luobing Research and Innovative Chinese Medicine, Shijiazhuang 050035, P.R China


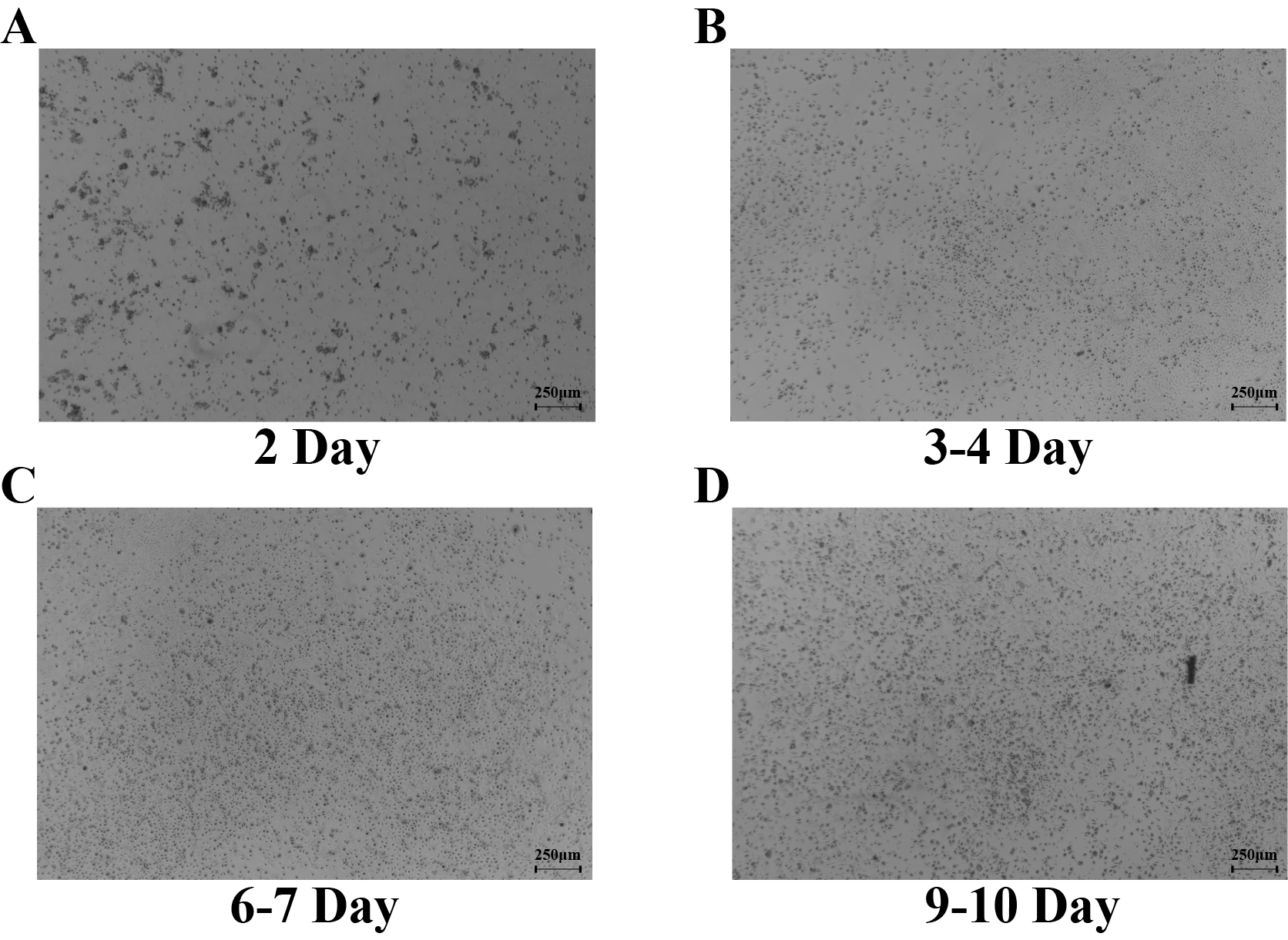


**Figure S1. Morphological observation of BMSCs in different stages.**


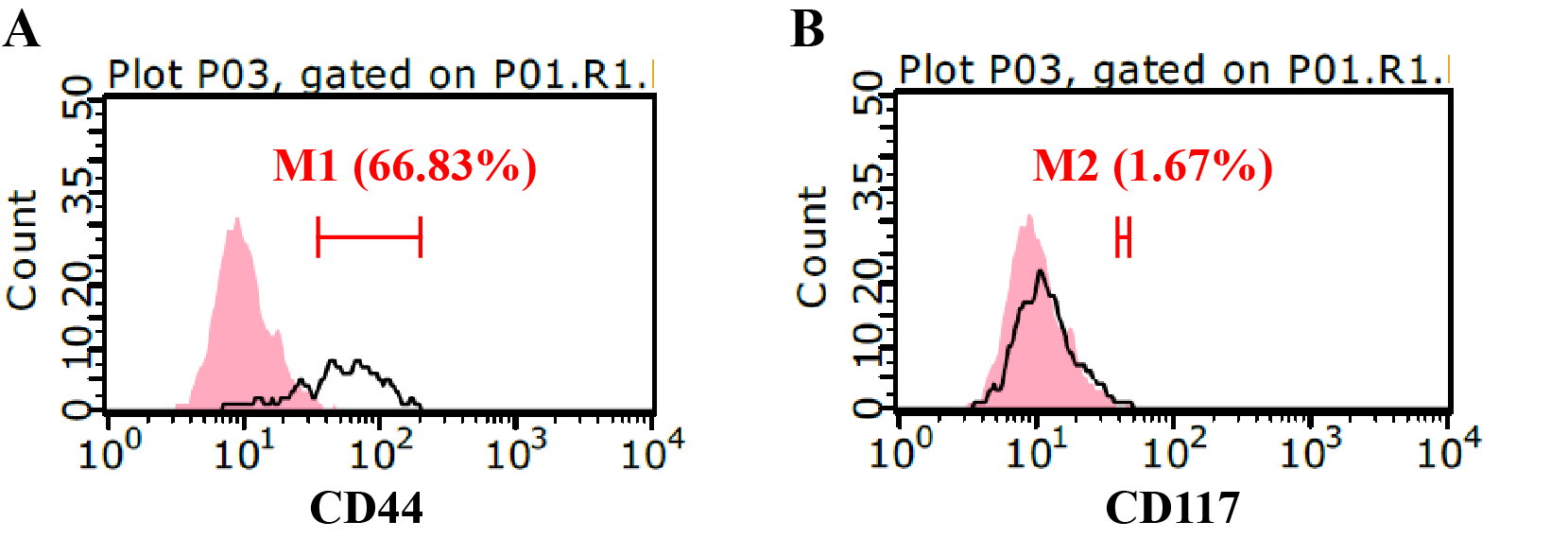


**Figure S2. Expression of surface markers in mouse BMSCs.**


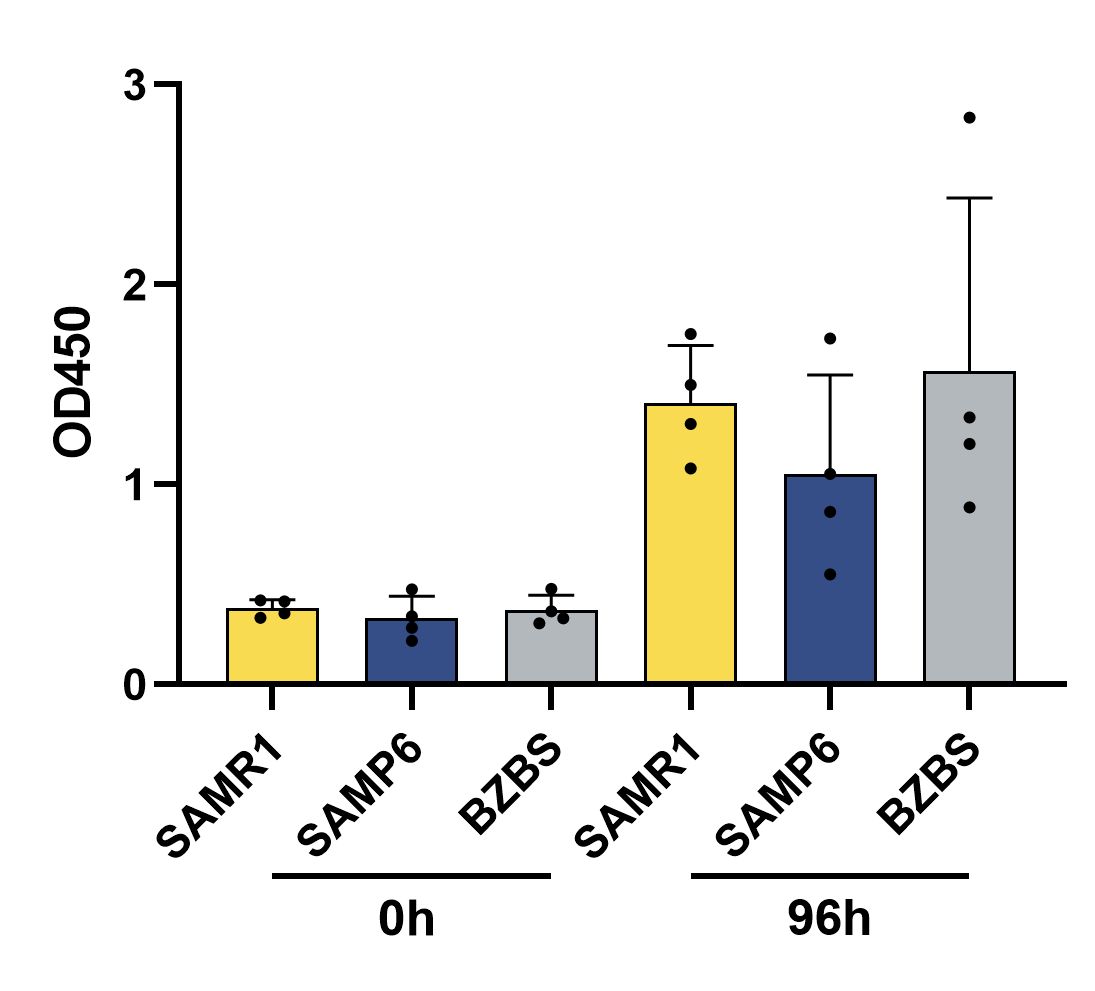


**Figure S3.** **Detection of viability of mouse BMSCs.**

**Table S1. The chemical components of Bazi Bushen.**

| **Chemical components** | **CAS** | **Molecular formula** | **Molecular weight (g/mol)** |
| --- | --- | --- | --- |
| Neochlorogenic acid | 906-33-2 | C_16_H_18_O_9_ | 354.31 |
| Chlorogenic acid | 327-97-9 | C_16_H_18_O_9_ | 354.31 |
| Cryptochlorogenic acid | 905-99-7 | C_16_H_18_O_9_ | 354.31 |
| Isoquercitrin | 21637-25-2 | C_21_H_20_O_12_ | 464.4 |
| Hyperin | 482-36-0 | C_21_H_20_O_12_ | 464.4 |
| Verbascoside | 61276-17-3 | C_29_H_36_O_15_ | 624.6 |
| Epimedin A | 110623-72-8 | C_40_H52O_19_ | 836.8 |
| Icariin | 489-32-7 | C_33_H_40_O_15_ | 676.7 |
| Baohuoside I | 113558-15-9 | C_37_H_30_O_10_ | 514.5 |
| Imperatorin | 482-44-0 | C_16_H_14_O_4_ | 270.28 |
| Osthole | 484-12-8 | C_15_H_16_O_3_ | 244.28 |
| Catalpol | 2415-24-9 | C_15_H_22_O_10_ | 362.33 |
| Schisandrin A | 61281-38-7 | C_24_H_32_O_6_ | 416.5 |
| Schisandrin B | 61281-37-6 | C_23_H_28_O_6_ | 400.5 |
